# A systematic review of adaptations of evidence-based public health interventions globally

**DOI:** 10.1186/s13012-018-0815-9

**Published:** 2018-09-26

**Authors:** Cam Escoffery, E. Lebow-Skelley, R. Haardoerfer, E. Boing, H. Udelson, R. Wood, M. Hartman, M. E. Fernandez, P. D. Mullen

**Affiliations:** 1 Rollins School of Public Health, Emory University, 1518 Clifton Road, Atlanta, GA 30322 USA; 2 University of Texas School of Public Health, 7000 Fannin, Ste 2522, Houston, TX 77030 USA

**Keywords:** Adaptation, Intervention, Modifications, Implementation, Evidence-based

## Abstract

**Background:**

Adaptations of evidence-based interventions (EBIs) often occur. However, little is known about the reasons for adaptation, the adaptation process, and outcomes of adapted EBIs. To address this gap, we conducted a systematic review to answer the following questions: (1) What are the reasons for and common types of adaptations being made to EBIs in community settings as reported in the published literature? (2) What steps are described in making adaptations to EBIs? and (3) What outcomes are assessed in evaluations of adapted EBIs?

**Methods:**

We conducted a systematic review of English language publications that described adaptations of public health EBIs. We searched Ovid PubMed, PsycINFO, PsycNET, and CINAHL and citations of included studies for adapted public health EBIs. We abstracted characteristics of the original and adapted populations and settings, reasons for adaptation, types of modifications, use of an adaptation framework, adaptation steps, and evaluation outcomes.

**Results:**

Forty-two distinct EBIs were found focusing on HIV/AIDS, mental health, substance abuse, and chronic illnesses. More than half (62%) reported on adaptations in the USA. Frequent reasons for adaptation included the need for cultural appropriateness (64.3%), focusing on a new target population (59.5%), and implementing in a new setting (57.1%). Common adaptations were content (100%), context (95.2%), cultural modifications (73.8%), and delivery (61.9%). Most study authors conducted a community assessment, prepared new materials, implemented the adapted intervention, evaluated or planned to evaluate the intervention, determined needed changes, trained staff members, and consulted experts/stakeholders. Most studies that reported an evaluation (*k* = 36) included behavioral outcomes (71.4%), acceptability (66.7%), fidelity (52.4%), and feasibility (52.4%). Fewer measured adoption (47.6%) and changes in practice (21.4%).

**Conclusions:**

These findings advance our understanding of the patterns and effects of modifications of EBIs that are reported in published studies and suggest areas of further research to understand and guide the adaptation process. Furthermore, findings can inform better reporting of adapted EBIs and inform capacity building efforts to assist health professionals in adapting EBIs.

**Electronic supplementary material:**

The online version of this article (10.1186/s13012-018-0815-9) contains supplementary material, which is available to authorized users.

## Background

Emphasis on evidence-based practice in medicine, public health, and the social services has led to a prominence of the application of practice guidelines and evidence-based interventions or EBIs. When situating an EBI in a new context, public health professionals, or health practitioners who work in community settings, sometimes adapt the EBI during the process of replication [1, 2]. However, in planning and implementing these interventions, there may be mismatches between the original EBI and the characteristics of the population of interest, implementing agency, and/or community [3]. In addition, agencies may lack the resources, funding, or expertise to deliver the EBI as it was originally intended [4]. Consequently, public health professionals often make both intended and unplanned program adaptations to the EBI to better fit the new audience or context.

The concept of program adaptation was originally introduced by Rogers when he defined adaptation as the degree to which an innovation is modified in the process of its adoption and implementation [5]. Other definitions have evolved in the era of translation of EBIs and the emergence of adaptation frameworks (Table 1). These definitions share similar characteristics, including modifying a program to meet the needs of the target population, local circumstances, or new contexts. Some definitions focus on the need to retain the core components or logic of the program [6–11]. The adaptations could be deletions, additions, or modifications [11]. Some posit that adaptations should be systematic or planned [12–14] to involve stakeholder input and to have a more rigorous process in program planning, while the CSAP (Center for Substance Abuse Prevention) framework notes that adaptations could be accidental modifications [11]. Moore and colleagues proposed the schema of timing of adaptation of proactive (planned) vs. reactive [15]. Furthermore, three definitions included modifications related to matching the culture for the new population, “cultural adaptation” [11, 16, 17]. A few definitions specify elements that could be changed such as program components, content, provider, and delivery [11, 14]. Of these definitions, CSAP’s Guidelines for Adaptation [11], Map of Adaptation Process [16, 18], ADAPT-ITT [9], and Research-based Program Adaptation [6] are cited most frequently in the published literature. In summary, although many adaptation definitions share similar characteristics, the most frequently cited ones do not emphasize the same concepts. Thus, it is important to discern how professionals in the field describe their adaptations, why they make modifications and the types of changes that they make, and how they use frameworks to conduct adaptations.

**Table 1:** Definitions of adaptation

Article	Adaptation definition
Backer (CSAP, 2002) [11]	“The deliberate or accidental modification of the program, including the following: a. Deletions or additions (enhancements) of program components; b. Modifications in the nature of the components that are included; c. Changes in the manner or intensity of administration of program components called for in the program manual, curriculum, or core components analysis; or d. Cultural and other modifications required by local circumstances.”
McKleroy et al. 2006 [16]	Quotes Rogers’ (1995) definition and the CSAP definition (see above).
Solomon et al. [6]	Modifying an efficacious program to meet the needs of its new target population and community context while retaining fidelity (or adherence) to its core components.
Smith and Caldwell [14]	“Evidence-based programs should not be changed randomly but should be modified based on a careful review of program content, the theoretical underpinnings involved, and the context of the new environment. Four different forms of adaptation need to be considered: structural, content, provider, and delivery.”
Wingood and DiClemente [9]	“The process of modifying an EBI without competing with or contradicting its core elements or internal logic.”
Barrera and Castro, Kumpfer et al. [17, 22]	Developing cultural adaptations or accommodations of EB practices for international transport is a … “process requiring careful assessment of the local political, religious, and economic context as well as the cultural norms and family practices of country and internal ethnic groups. It should be a careful and rigorous process …guided by research and theory.”
Lee et al. [7]	“Inherent in [the process of moving evidence-based programs (EBPs) from research to practice] is the tension between implementing programs with fidelity and the need to tailor programs to fit the target population.”
Card et al. [3]	“The process of altering a program to reduce mismatches between its characteristics and those of the new context in which it is to be implemented or used.”
Chen et al. [8]	“Methods of planned adaptation identify differences in the new target population and attempt to make changes to the EBI that accommodate these differences without diluting the program’s effectiveness.”
Rolleri [10]	“The process of making changes to a program in order to make it more suitable for a particular population or for an organization, based upon its capacity. Changes to a program should be made without compromising or deleting the program’s core components.”
Bartholomew et al. [42]	Systematic adaptation requires that planners make adaptation decisions by comparing the logic of change in the EBI with the needs of the new community. Planners should only make changes that correspond with mismatches between the EBI and community needs.

Previous reviews have found that modifications to original EBIs often occur spontaneously when they are adopted into other practice settings [1, 15]. Common reasons for adaptations include responding to participants’ attributes [18, 19], needs [20] or culture [15], constraints such as limited time or resources [15, 19–21], issues related to participant recruitment or retention [15], and accommodating practice or setting circumstances/context [20].

Increased development of models and frameworks to guide the adaptation of EBIs began with national EBI dissemination efforts related to disease prevention areas in substance abuse and HIV/AIDS [6, 9, 14] or cultural adaptations to existing programs [7, 22]; these frameworks provide approaches to facilitate adaptation. Escoffery and associates recently conducted a scoping study that found 13 adaptation frameworks [23]. They reported 11 common steps including assess the community, understand the intervention, select intervention, consult with experts, consult with stakeholders, decide what needs adaptation, adapt the original program, train staff, test the adapted materials, implement, and evaluate. These frameworks enhance the translation of evidence-based practices. As Wandersman’s Interactive Systems Framework suggests, supports are necessary to guide the public health system or agencies to adopt and implement new public health interventions [24]. These frameworks assist public health professionals as capacity building tools for a structured adaptation process.

Limited research has explored *how* adaptation occurs in practice. Little is understood about who is involved in adaptation processes, what common types of changes are made to the original program, and what mechanisms are used. This review advances the concept of adaptation and elucidates common adaptation processes in real-world community settings as reported in the published literature. Community settings are defined as various organizations or places in communities such as schools, faith-based organizations, social services or public health agencies, households, or worksites. The research questions for the review were as follows:

1. What are the reasons for and common types of adaptations being made public health EBIs in community settings as reported in the published literature?
2. What steps are described to making adaptations to EBIs?
3. What outcomes are assessed in evaluations of adapted EBIs?

## Methods

We followed procedures for systematic reviews based on the Cochrane Handbook of Systematic Reviews of Public Health Interventions [25] and the reporting guideline, Preferred Reporting Items for Systematic Reviews and Meta-Analyses (PRISMA) [26].

### Search strategy

We searched Ovid PubMed, PsycINFO, PsycNET, and CINAHL with the assistance of an experienced health sciences librarian. The date of the last search was December 2015. Concepts for the search included adaptation, evidence-based interventions and practice, health behavior, and quality of healthcare*.* Combinations of the associated MeSH terms were used to develop the initial PubMed search and then adapted to search other databases. The search strategies can be found in Additional file 1. We also manually cross-referenced reference lists of included studies. We downloaded relevant citations into a reference manager software program, EndNote, which facilitated removing duplicate citations identified in the multiple databases. We exported the resulting composite library into an Excel file for documentation of the title and abstract review process.

### Eligibility criteria

The project team created an Eligibility Assessment Checklist restricting included articles to those reporting primary studies published in English after 1995 and that examined the adaptation process or outcomes of an adapted evidence-based intervention (public health program or policy). Programs are defined as a combination of strategies designed to create behavior change or improve health status and policies are rules, regulations, or actions related to a health goal or service. These adaptations reported could be proactive (purposeful) or reactive. Articles were excluded if they did not describe the adaptation methods or if the full-text article was unable to be located after an exhaustive search. We combined articles reporting different aspects of the same EBI, e.g., the evaluation findings and the adaptation process.

### Screening

Two trained reviewers (CE, HU) independently screened titles and abstracts after duplicates were removed, using the Eligibility Assessment Checklist. We selected potentially relevant abstracts for a full-text review conducted independently by the two trained reviewers. The first author resolved any disagreement between the reviewers.

### Study quality assessment

We assessed study quality of the articles based on their use of a theory or adaptation framework, and in the case of those that included an evaluation, we assessed the rigor of the design. We used these variables descriptively, however, and did not differentiate studies based on these variables.

### Data abstraction and analysis

We reviewed the articles of EBI adaptations for six categories of variables: (1) characteristics of the original and adapted EBI (name, disease/topic, population and setting), (2) reason(s) for adaptation, (3) type(s) of modifications, (4) steps (tasks) in adaptation described by the authors, (5) reference to an adaptation framework, and (6) measures of implementation and intervention outcomes (see definitions in Additional file 2). In addition, we described how they presented the EBI adaptations made in the articles. We used a structured data abstraction form designed in Excel 2016 to record the extracted information. We used Stirman and associates’ typology of modifications [1], the adaptation steps identified in the scoping study of adaptation frameworks [23], and implementation outcomes defined by Proctor and colleagues [27]. Context modifications were defined as changes to format, location, or personnel delivering the intervention, while content modifications were changes to the intervention materials, procedures, or delivery. In coding adaptation steps, we combined consulting with stakeholders and experts and had an “other” option, resulting in nine named steps. For each study, we examined bias in the study through documentation of participants (e.g., selection, generalizability), study design, and inadequate results reporting. Two trained reviewers (CE, HE, RW, ME, PDM, EL) independently coded the included articles. Discrepancies were discussed and adjudicated by the larger team.

### Data synthesis and presentation

We presented summaries of study-specific adaptation reasons, steps, types of adaptations, and outcome measures with descriptive statistics across studies. We described the original and adapted EBI, the study population, reasons for adaptations, the name of adaptation frameworks, and examples of adaptations qualitatively.

## Results

**Fig. 1:**
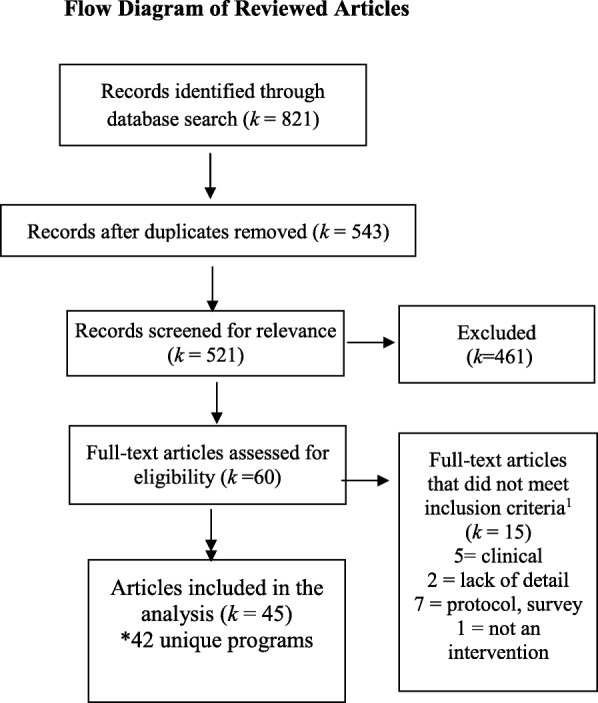
Flow diagram of reviewed articles

We found 543 unique citations that yielded 45 articles reporting 42 distinct program adaptations after the two levels of screening (Fig. 1). Main reasons for exclusion were a lack of description of the adaptation process or methods, not being a public health program or policy, and not being a primary study (e.g., protocol, review).

### Adaptation characteristics

Publication years of the primary citations are from 2003 to 2014, and common disease topics included HIV/AIDS, mental health, substance abuse, and chronic illnesses (Table 2). Many of these studies had non-experimental designs (*k =* 27, 64.3%), and the remainder had experimental (*k =* 12, 28.6%) or quasi-experimental (*k =* 3, 7.1%) designs. Thirty-six adaptations included an evaluation. Most (*k =* 26, 61.9%) reported on adaptations that took place in the USA, and one EBI was adapted in three locations (USA, Africa, and Asia). Other EBIs were adapted in Africa (*k =* 4), Asia (*k =* 5), Europe (*k =* 3), Canada (*k =* 1), the Caribbean (*k =* 1), and Australia (*k =* 1).

**Table 2:** Characteristics of included reports, reasons for adaptation, and frameworks

First author, year	Original EBI name (adapted EBI name) study design	EBI disease/topic	Reason for adaptation	Target population/setting		Adaptation framework (if mentioned)
				Original (author)	Adapted	
Reijneveld, 2003 [53]	Healthy & Vital (no change) Experimental—RCT	Physical inactivity/poor physical and mental well-being	Cultural appropriateness New population	People aged ≥ 65 in welfare services in the Netherlands	Turkish immigrants aged ≥ 45 in welfare services in the Netherlands	N/R
Komro, 2004 [54]	Project Northland (Project Northland Chicago) Experimental—RCT	Alcohol use	Cultural appropriateness New population New community	Mostly white, 6th–8th grade students in rural NE Minnesota, USA	Culturally diverse 6th–8th grade students in Chicago, IL, USA	N/R
Sarkisian, 2005 [55]	Empowerment: Facilitating a Path to Personal Self-Care (N/R) Non-experimental	Diabetes	Cultural appropriateness New population	Younger, mostly Caucasian patients with diabetes in the USA	African Americans and Latinos aged ≥ 55 with diabetes in public health diabetes and geriatrics clinics and senior centers in South Los Angeles, CA, USA	N/R
Tsey, 2005 [56]	Family Wellbeing (no change) Non-experimental	Teasing, bullying, fighting, low self-esteem, truancy	New population New community	Adults in Aboriginal Australia (Tsey [57])	Students in 2 primary schools in remote indigenous communities in Cape York Peninsula, far north Queensland, Australia	N/R
Villarruel, 2005 [58]	Be Proud! Be Responsible! (¡Cuídate!) Experimental—RCT	HIV	Cultural appropriateness New population	African American adolescents aged 13–18 from community-based agencies in Philadelphia, USA	Inner-city Latino adolescents aged 10–19 in Philadelphia, PA, USA	N/R
Belanksy, 2006 [59]	Integrated Nutrition Education Program, INP (Integrated Nutrition and Physical Activity Program, INPAP) Non-experimental	Nutrition and physical activity	Cultural appropriateness New community	Elementary school children in a school setting in Denver, USA	2nd and 3rd grade students in a rural, biethnic, low-income county in south-central Colorado, USA	N/R
Hitt, 2006 [60]	Project RESPECT (N/R) Non-experimental	HIV/STD	New population New community	Heterosexual individuals aged ≥ 14 attending 5 public STD clinics in the USA (Kamb [61])	MSM, IDU, and heterosexual individuals attending either a local health department or a CBO for prevention counseling services in Texas, USA	N/R
Somerville, 2006 [62]	Popular Opinion Leader, POL (Young Latino *Promotores*, YLP) Non-experimental	HIV	Cultural appropriateness New population	White gay men in gay venues frequented predominantly by whites in midsized southern cities in the USA	Latino migrant MSM aged 18–30 in Texas and California USA-Mexico border communities	N/R
NIMH Collaborative HIV/STD Prevention Trial Group, 2007 [38]	Community Popular Opinion Leader (C-POL) (no change) Experimental—RCT	HIV/STD	Cultural appropriateness New community	Populations vulnerable to HIV risk behavior in the USA	Individuals aged 18–49 at food markets with individually owned stalls in Fuzhou, China and individuals aged 18–30 in the following settings; wine shops in slums in Chennai, India; gathering points of young, high-risk people in barrios in Lima, Chiclayo, and Trujillo, Peru; trade school dorms in St. Petersburg, Russia; and retail establishments in rural Zimbabwe	N/R
Tsarouk, 2007 [63]	Reconnecting Youth (RY) (no change) Non-experimental	Substance abuse and HIV transmission	Cultural appropriateness	High-risk students aged 14–18 in the USA	Russian adolescents aged 14–17 with poor school performance and mild behavioral problems in schools in Moscow, Russia	N/R
Beattie, 2008 [64]	Swim and Survive, and Infant Aquatics (Water Safety in the Bush) Non-experimental	Water safety/drowning	Cultural appropriateness New community	Infants, children 5–14 years, and parents in Australia	Children and adults in rural and remote Australian communities	N/R
Cornelius, 2008 [28]	Sisters Informing Sisters on Topics about AIDS, SISTA (Women Informing Women on Topics about AIDS, WIWTA) Non-experimental	HIV/AIDS	New population New community	Young African American girls in heterosexual relationships in San Francisco, USA (DiClemente [65])	African American women ≥ 50 in heterosexual relationships who frequent churches located in low-income areas of North Carolina, USA	N/R
Gitlin, 2008 [30]	Chronic Disease Self-Management Program, CDSMP (Harvest Health, HH)	Chronic disease self-management	New population New community	Middle-class white patients aged ≥ 40 in community-based sites in the USA (Lorig [66])	African Americans aged ≥ 60 with chronic condition(s) in a senior setting in Philadelphia, PA, USA	N/R
Lerdboon, 2008 [67]	Vietnamese Focus on Kids (Exploring the World of Adolescents, EWA) Non-experimental	HIV/AIDS	Cultural appropriateness New community	Adolescents in Khanh Hoa Province, Vietnam	Adolescents aged 15–21 in both rural and urban Vietnam	N/R
Steiker, 2008 [29]	Keepin’ It REAL (Refuse, Explain, Avoid, and Leave) (N/R) Quasi-experimental	Substance abuse prevention	Cultural appropriateness New population New community	Middle school youth in the USA	Adolescents aged 14–19 in high risk, unique community settings in Texas, USA	Castro - cultural adaptation
Burgio, 2009 [68]	Resources for Enhancing Alzheimer’s Caregiver Health, REACH II (REACH OUT, Offering Useful Treatments) Non-experimental	Alzheimer’s disease	Implementation ease/feasibility	In-home Alzheimer’s caregivers in USA cities	Alzheimer’s caregivers in Area Agencies on Aging in Alabama, USA	N/R
Fiscian, 2009 [69]	Making Proud Choices (N/R) Non-experimental	HIV/AIDS	Cultural appropriateness New population New community	Minority adolescents in the USA	Adolescent girls aged 10–14 in a church-affiliated junior secondary school in Ghana	N/R
Mueller, 2009 [31]	*¡Cuídate!* (no change) Non-experimental	HIV/AIDS	New community	Latino youth aged 13–18 in Northeast Philadelphia schools, USA	Latino youth in a urban high school in Denver, CO, USA	N/R
Pekmezi, 2009 [70]	Individually tailored physical activity print intervention (*Seamos Activas*) Experimental—RCT	Physical inactivity and related chronic illnesses	Cultural appropriateness New population	Sedentary adults in the USA	Overweight/obese Latinas aged 18–65 with low income and acculturation in Providence, RI, USA	N/R
Stevens, 2009 [71]	REACH II (Support Teams for Caregivers) Non-experimental	Alzheimer’s disease or dementia	Implementation ease/feasibility	Family caregivers of patients with Alzheimer’s disease or dementia in 5 USA cities	Family caregivers of patients with Alzheimer’s disease or dementia in Texas, USA	RE-AIM and REP
DePue, 2010 [72]	Project Sugar 2, PS2 (Diabetes Care in American Samoa) Experimental—RCT	Type 2 diabetes	Cultural appropriateness New population	Urban African Americans aged ≥ 25 with diabetes in Baltimore, USA	Individuals aged ≥ 21 with type 2 diabetes in American Samoa	Lau’s framework for cultural adaptation
Domenech Rodriguez, 2011 [73]	Parent Management Training—Oregon Model, PMTO (*Criando con Amor: Promoviendo Armonía y Superación*, CAPAS) Experimental—RCT	Parenting	Cultural appropriateness New population	Divorcing mothers with sons in 1st–3rd grades in a medium-sized city in the Pacific NW, USA (Forgatch [74])	Spanish-speaking Latino parents or relatives who co-parent in rural Utah, USA	CAP and EVM
Poulsen, 2010; [39] Vandenhoub, 2010 [75]	Parents Matter! (Families Matter!) Non-experimental	HIV	Cultural appropriateness New community	African American parents of preteens aged 9–12 in a controlled clinical setting in the USA	Families with children aged 9–12 in Asembo, rural west Kenya	MAP
Sadler, 2010 [76]	“Cancer Clinical Trials: The Basics” and “*Conversemos un rato: Información para combatir el cáncer en su comunidad*” (N/R) Non-experimental	Breast cancer	Cultural appropriateness New population	Individuals with cancer in the USA	African American/Hispanic American women, or women from diverse communities with breast cancer in California, USA	N/R
Rotheram-Borus, 2011 [77]	Project TALC (LA Project TALC in Los Angeles, Family to Family in Thailand, Mentor Mothers in South Africa) Non-experimental	HIV	Cultural appropriateness New community	Parents living with HIV and their children or caregiver supports in New York City, USA	Parents living with HIV and their children or caregiver supports in the USA (Los Angeles, CA), Thailand, and South Africa	CQI
Cardona, 2009 [78]	Parent Management Training—Oregon Model, PMTO (N/R) Non-experimental	Parenting/mental health	Cultural appropriateness New population New community	Divorcing mothers with sons in 1st–3rd grades in a medium-sized city in the Pacific Northwest, USA (Forgatch [74])	Latino immigrant parents with children aged 6–12 with mild behavioral problems in Detroit, MI, USA	EVM
Feinberg, 2012 [79]	Problem-Solving Treatment (Problem-Solving Education) Experimental—RCT	Depression	New population	Adults with depression in general practices in Oxford, United Kingdom (Gath [80])	Mothers with limited incomes and high rates of depression in 3 settings where they receive services in Massachusetts, USA	Backer’s 6-step approach Castro’s cultural adaptation
Parker, 2012; [81] Chen, 2013 [82]	Arthritis Self-Help Program, ASHP (no change) Non-experimental	Arthritis	Cultural appropriateness New population Condense program	Younger, mostly non-Hispanic white adults in the USA	African American, Hispanic, and non-Hispanic white older adults attending senior centers in New York City, NY, USA	M-PACE
Reid, 2012 [83]	Cognitive Behavioral Stress Management (CBSM) (no change) Non-experimental	Substance abuse, sexual behavior, and HIV	Cultural appropriateness New community	Drug abusers	HIV-positive substance abusers in recovery in Trinidad and Tobago	N/R
Rosati, 2012 [84]	Family Matters (Thai Family Matters) Experimental—RCT	Alcohol, tobacco, and other drug use	Cultural appropriateness New community	Parents and children in the USA	Adolescents aged 13–14 and their parents in Bangkok, Thailand	N/R
Tomioka, 2012 [85]	Chronic Disease Self-Management Program, CDSMP (*Ke Ola Pono*) Non-experimental	Chronic disease self-management	Cultural appropriateness New population	Adults aged ≥ 40 with chronic diseases in community-based sites in California, USA (Lorig [66])	Asians and Pacific Islanders with chronic diseases in Hawaii, USA	CDC’s adaptation traffic light
Danielson, 2013 [33]	Sistas Informing, Healing, Living, and Empowering, SiHLE (SiHLEWeb) Non-experimental	HIV/STD	Cultural appropriateness Implementation ease/feasibility	African American adolescents in community health agencies in the USA (DiClemente [65])	Community-dwelling traditionally underserved African American girls aged 13–18 in the Southeast USA	N/R
Fasula, 2013 [86]	Project Safe (Project POWER) Non-experimental	HIV/STD	New population New community	African American and Mexican American women in STD clinics in in San Antonio, USA (Shain [87])	HIV-negative women with sentences up to 14 months due to be released within 6 months in North Carolina women’s prison facilities, USA	MAP
Parker, 2013a; [88] Parker; 2013b [89]	Healthy Living Project (Supporting Youth and Motivating Positive Action, SYMPA) Non-experimental	HIV/AIDS	New population New community	Adults living with HIV in the USA	Youth aged 15–24 living with HIV/AIDS in Kinshasa, Democratic Republic of the Congo	ADAPT
Wainer, 2013 [90]	Reciprocal imitation training (RIT) (no change) Non-experimental	ASD	Implement in new community setting Make program more widely accessible	Individuals working with children with ASD, including parents, in the USA	Individuals working with children with ASD, including parents, in the participants’ homes and research lab in the Midwestern USA	N/R
Williams, 2013 [91]	Adherence Through Home Education and Nursing Assessment, ATHENA (N/R) Experimental—RCT	HIV/AIDS	Cultural appropriateness New population New community	European, African and Hispanic individuals with a high prevalence of substance abuse and mental illness for whom ARV therapy was prescribed in the northeastern USA	Patients living with HIV/AIDS receiving ARV therapy from the Hunan China CARES clinical program in rural south central China	Castro’s cultural adaptation
Baydala, 2014 [92]	Life Skills Training, LST (*Nimi Icinohabi*) Quasi-experimental design	Substance abuse	Cultural appropriateness New population	Elementary, middle, and high school students, including ethnic minority youth in the USA	Aboriginal school-age children in Central Alberta, Canada	N/R
Broning, 2014 [93]	Strengthening Families Program for Parents and Youth 10–14, SFP 10–14 (*Familien Stärken*) Experimental—RCT	Substance abuse	Cultural appropriateness New community	Adolescents aged 10–14 and their caregivers in rural economically deprived regions in Iowa, USA	Adolescents aged 10–14 and their caregivers in socially deprived urban districts in Hamburg, Schwerin, Hanover and Munich, Germany	N/R
Cariou, 2014 [94]	Pool Cool (no change) Non-experimental	Skin cancer	New population Implementation ease/feasibility	Aquatics instructors, kids aged 5–10, parents and other pool users in Hawaii and Massachusetts, USA (Glanz [95])	Children and adolescents aged 2–17 enrolled in swim lessons at the Payette Municipal Pool, rural Idaho, USA	N/R
Reback, 2014 [96]	Gay-specific cognitive behavioral therapy, GCBT (Getting Off: A Behavioral Treatment Intervention for Gay and Bisexual Male Methamphetamine Users) Experimental - RCT	Methamphetamine use/HIV	New community	Methamphetamine-using gay and bisexual men in a controlled clinical setting in the USA	Methamphetamine-using MSM in a community-based HIV prevention setting in Los Angeles, CA, USA	N/R
Riggs, 2014 [32]	Family Overweight: Comparing Use of Strategies, FOCUS (Family Wellness Program, FWP) Non-experimental	Pediatric obesity	Implementation ease/feasibility New community	Obese children and their parents in the USA (Saelens [97])	Obese children aged 6–12 and their parents in primary care clinics near Seattle, WA, USA	N/R
Tu, 2014 [98]	Clinic-based educational program to promote CRC screening among Chinese immigrants (N/R) Quasi-experimental	Colorectal cancer screening	New population Implementation ease/feasibility	Chinese immigrant in a community health center in the metropolitan area of Seattle, USA (Tu [99])	Vietnamese patients of community health centers in the metropolitan area of Seattle, WA, USA	Diffusion of innovations theory

*N/R* not reported, *ASD* autism spectrum disorder, *ARV* antiretroviral, *CAP* cultural adaptation process, *CBO* community-based organization, *CQI* continuous quality improvement, *EVM* ecological validity model, *IDU* injection drug user, *M-PACE* Method for Planned Adaptation through Community Engagement, *MAP* Map of Adaptation Process, *MSM* men who have sex with men, *RE-AIM* Reach, Effectiveness, Adoption, Implementation and Maintenance, *REP* Replicating Effective Programs, *STD* sexually transmitted disease

### Reasons for adaptation

The most common reasons for adaptation included the need for a culturally appropriate program (*k =* 27; 64.3%), a new target population (*k =* 25; 59.5%), and a new community setting (*k =* 24; 57.1%) (Table 2). Less common reasons for adaptation were the desire to improve ease and feasibility of implementation (*k =* 6; 14.3%), attempting to make the program more widely accessible (*k =* 1; 2.4%), and trying to condense the original intervention (*k =* 1; 2.4%).

### Types of modifications

Authors reported making an average of 3.4 (SD = 0.90, range 2–5) different types of adaptations with a mode of 3 (Table 3). All 42 (100%) reported some modification of the EBI content. The form this took usually included tailoring (*k =* 39; 92.9%) or adding elements (*k =* 30; 71.4%). For example, Cornelius and associates modified HIV prevention videos originally tested with young pregnant women to be relevant to older African American women [28]. In the adaptation reported by Steiker, the study team added four new videos to accompany the curriculum and rewrote scenarios used in the workbooks to incorporate local culture [29]. In the EBI adapted by Gitlin and associates, a moment of silence was added at the beginning of each session to recognize spiritual practices and their importance to participants [content modification-adding elements [30]]. More than half of the authors reported shortening the original EBI as one of the adaptations made. For the 42 programs, some teams described adapting the intervention content by shortening it (*k =* 13; 31.0%), removing elements (*k =* 12; 28.6%), loosening the structure (*k =* 10; 23.8%), lengthening the program (*k =* 9; 21.4%), substituting modules or activities (*k =* 7; 16.7%), or integrating other approaches to the intervention (*k =* 5; 11.9%).

**Table 3:** Summary of adaptation characteristics reported in peer-reviewed literature (EBIs), *k* ***=*** 42

Adaptation characteristics	Studies reporting characteristic *k* (%)
Type of modification	
Content	42 (100%)
Tailoring	39 (92.9%)
Adding elements	30 (71.4%)
Shortening	13 (31.0%)
Removing elements	12 (28.6%)
Loosening structure	10 (23.8%)
Lengthening	9 (21.4%)
Substitution	7 (16.7%)
Integrating other approach	5 (11.9%)
Reorder elements	4 (9.5%)
Integrating intervention	2 (4.8%)
Departing	2 (4.8%)
Repeating elements	1 (2.4%)
Cultural modification	31 (73.8%)
Context	40 (95.2%)
Population	33 (78.6%)
Setting	29 (69.0%)
Other	3 (7.1%)
Delivery	26 (61.9%)
Deliverer	16 (38.1%)
Mode/medium	14 (33.3%)
Other	4 (9.5%)
Training	16 (38.1%)
Evaluation	19 (45.2%)
Change to core elements	4 (9.5%)
Reasons for adaptation	
Cultural appropriateness	27 (64.3%)
Focus on new target population	25 (59.5%)
Implement in new community setting	24 (57.1%)
Improve ease and feasibility of implementation	6 (14.3%)
Make program more widely accessible	1 (2.4%)
Condense program	1 (2.4%)
Outcomes	
Implementation	
Acceptability	28 (66.7%)
Fidelity	22 (52.4%)
Feasibility	22 (52.4%)
Adoption	20 (47.6%)
Sustainability	11 (26.2%)
Other	5 (11.9%)
Behavioral/program	
Behavior	30 (71.4%)
Practice	9 (21.4%)
Knowledge	7 (16.7%)
Self-efficacy	5 (11.9%)
Environment	4 (9.5%)
Well-being/health	3 (7.1%)
Attitudes	3 (7.1%)
Skills	3 (7.1%)
Communication	2 (4.8%)
Policy	0
Other	4 (9.5%)
Individual satisfaction	11 (26.2%)

Nearly all of the adaptations (*k =* 40; 95.2%) described modifying context, and 31 (73.8%) included cultural modifications. Most context modifications included making changes to the original EBI by adapting it to fit with the new intervention population (*k =* 33; 78.6%) and setting (*k =* 29; 69.0%). Mueller and colleagues, for example, adapted a curriculum originally delivered in community agencies and after-school programs to a school setting [31]. Over half of the adaptations included changes to the delivery of the original intervention (*k =* 26; 61.9%), either by modifying the role of the personnel delivering the intervention (*k =* 16; 38.1%) or by adapting the format or channel of delivery (*k =* 4; 33.3%). Masters-level research interventionists, for instance, delivered the family-based behavioral pediatric obesity treatment rather than medical staff in the intervention reported by Riggs and colleagues [32]. In the EBI adapted by Danielson and team, a web-based delivery platform was used instead of small group sessions [33]. Fewer authors reported modifying procedures for training personnel (*k =* 16; 38.1%) or for evaluating the program (*k =* 19; 45.2%). Four (9.5%) studies described changing what they regarded as core elements of the original EBI.

### Patterns of adaptation types

**Fig. 2:**
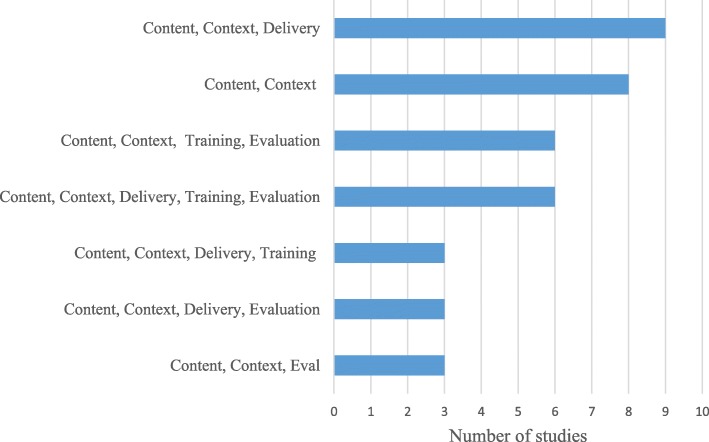
Common patterns of types of adaptations across studies

The most common combinations were content, context, and delivery (*k =* 9), and content and context (*k =* 8) (Fig. 2). Content and context were part of all other combinations; four other combinations only had one study each.

For content adaptations, the classifications reported varied greatly. However, some patterns emerged with certain content combinations, including tailoring, adding elements, and cultural modifications (*k =* 5); tailoring, adding elements, loosening structure, and cultural modifications (*k =* 4); tailoring, adding elements, lengthening, and cultural modifications (*k =* 3); and tailoring and cultural modifications.

### Steps in adaptation

**Fig. 3:**
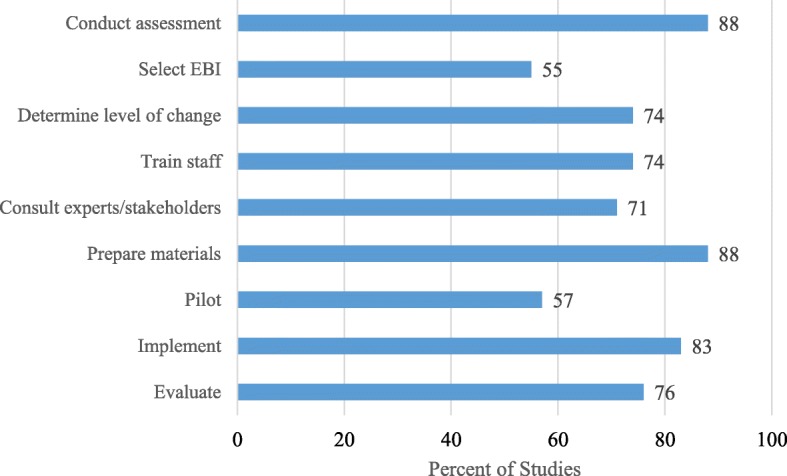
Steps taken in the adaptation process across studies

Each of the nine steps derived from the scoping review of evaluation frameworks [23] is represented in most of the adaptations (combining consulting with experts and stakeholders), with fewer reporting selecting the EBI (*k =* 23; 54.8%) and pilot testing (*k =* 24; 57.1%) (Table 3); 37 (88.1%) conducting a community assessment; 37 (88.1%) preparing new materials; 35 (83.3%) implementing the adapted intervention; 32 (76.2%) evaluating the adapted intervention; 31 (73.8%) determining needed changes based on action step assessments; 31 (73.8%) training staff members; and 30 (71.4%) consulting stakeholders or experts before adapting the materials (Fig. 3). Overall, the average number of steps was 6.7 (range 3–9, mode = 7). Of the 37 authors who reported conducting community assessments, 21 (56.8%) held focus groups with community members, 12 (32.4%) conducted interviews with key informants and stakeholders, five (13.5%) formed and consulted with community advisory boards or steering committees, and two (5.4%) administered a survey to get community feedback. Ten of these (27.0%) used a combination of methods to collect community input and assess need.

### Use of adaptation frameworks

The authors of less than half of the reports named a pre-existing adaptation framework as guiding the adaptation process (*k* = 15; 35.7%) (Table 2). Most frameworks were mentioned once; the Ecological Validity Model, Map of the Adaptation Process, and Cultural Adaptation Framework were referenced twice.

### Intervention outcomes

Of the 36 reports that included an evaluation, most authors reported measuring program acceptability (*k =* 28; 66.7%), fidelity (*k =* 22; 52.4%), and feasibility (*k =* 22; 52.4%) (Table 3). With respect to implementation outcomes, most authors reported evaluating program acceptability (*k =* 28; 66.7%), fidelity (*k =* 22; 52.4%), and feasibility (*k =* 22; 52.4%). Several studies also reported assessing the adoption/implementation (*k =* 20; 47.6%) and sustainability of the program (*k =* 11; 26.2%). Numerous authors also reported measuring behavioral and program outcomes. The majority reported measuring behavioral outcomes (*k =* 30; 71.4%), while a smaller number measured changes in practice (*k =* 9; 21.4%), knowledge (*k =* 7; 16.7%), self-efficacy (*k =* 5; 11.9%), or environment (*k =* 4; 9.5%). Only a few studies included assessments of changes in well-being (*k =* 3; 7.1%), attitudes (*k =* 3; 7.1%), skills (*k =* 3; 7.1%), or communication (*n* = 2; 4.8%). Lastly, 11 (26.2%) of the evaluations included satisfaction with the adapted intervention as an outcome (Table 4).

**Table 4:** Characteristics of the adaptations (*k* = 42)

First author, year	Adaptation type^1^					Specific modifications			Adaptation steps^2^										Evaluation outcomes (*k* = 36)	Modification/adaptation example
	1	2	3	4	5	Content	Context	Delivery	1	2	3	4	5	6	7	8	9	10		
Reijneveld, 2003 [53]	x	x	x	x	x	Tailoring Adding elements Lengthening Substitution Cultural modification	Population	Deliverer Other	x	x		x	x	x	x	x	x		Acceptability Fidelity	Examples regarding safety excluded cycling because few Turkish immigrants cycle
Komro, 2004 [54]	x	x	x			Tailoring Adding elements Cultural modification	Setting Population	Mode/medium Deliverer	x	x	x	x	x	x	x	x			Acceptability Fidelity	Audiotape vignettes re-taped with African American and Hispanic actors
Sarkisian, 2005 [55]	x		x			Tailoring Adding elements Lengthening Cultural modification	Setting Population	–	x		x		x						Acceptability	Expanded focus to more explicitly include family members
Tsey, 2005 [56]	x	x	x			Tailoring Shortening Loosening structure	Setting Population	Mode/medium Deliverer	x							x	x		Acceptability Adoption Sustainability Individual satisfaction	Students interviewed their role models, explaining why they looked up to that person
Villarruel, 2005 [58]	x	x		x	x	Tailoring Adding elements Cultural modification	Population	–	x	x	x	x	x	x	x	x	x		Acceptability Individual satisfaction	Presented the view of machismo that incorporated the values of caring for and protecting others, so condom use could be presented as consistent with machismo
Belanksy, 2006 [59]	x	x		x	x	Tailoring Adding elements Shortening Cultural modification	Setting Population	–	x		x	x	x	x	x	x	x		–	Lessons simplified so that they could be completed during the 1-h classroom period
Hitt, 2006 [60]	x	x		x		Tailoring Loosening structure	Setting Population	–	x	x	x	x	x	x		x	x		Acceptability Adoption Fidelity Feasibility Sustainability Individual satisfaction	Intervention protocols and supporting materials (tools) were tailored for local circumstances
Somerville, 2006 [62]	x	x		x	x	Tailoring Adding elements Substitution Integrating other approach Cultural modification	Setting Population	–	x	x	x	x	x	x	x	x	x		Acceptability Fidelity Other Individual satisfaction	A variety of successful Latino-focused HIV prevention training programs were integrated into the adapted intervention
NIMH Collaborative HIV/STD Prevention Trial Group, 2007 [38]	x	x		x	x	Tailoring Cultural modification	Other	–	x	x	x	x	x	x	x	x	x	x	Adoption Fidelity Feasibility Sustainability	Specific messages used in training were based on findings that emerged from the ethnography with each site’s populations
Tsarouk, 2007 [63]	x	x				Tailoring Removing elements Shortening Substitution Cultural modification	Other	–	x		x		x	x	x		x		Acceptability Feasibility Individual satisfaction	Some of the support behaviors, such as applauding in response to a group member’s participation, were removed because teens said that it is not a natural expression of support in this informal situation
Beattie, 2008 [64]	x	x			x	Tailoring Removing elements Shortening Integrating other approach Cultural modification	Setting Population	–	x		x	x	x			x	x		Acceptability Adoption Sustainability	Some sites used a swim camp model, with several days of training provided often on two or three occasions and typically at a central point for families traveling long distances
Cornelius, 2008 [28]	x	x				Tailoring Adding elements Removing elements Lengthening Substitution	Setting Population	–	x	x				x					–	Used videos that included information about HIV in older women
Gitlin, 2008 [30]	x	x	x			Tailoring Adding elements Cultural modification	Setting Population	–	x		x	x		x		x	x		Acceptability Adoption Fidelity Feasibility Sustainability Other Individual satisfaction	Introduction of moment of silence at the beginning of each session to recognize spiritual practices and their importance to participants
Lerdboon, 2008 [67]	x	x				Tailoring Adding elements Integrating other approach Cultural modification	Setting	–	x	x	x	x	x	x	x	x	x		Acceptability Adoption Feasibility	Gender-specific components were integrated throughout the curriculum through a story line about an adolescent boy and girl growing up in a fictional Vietnamese family, as well as gender-specific scenarios, activities and messages
Steiker, 2008 [29]	x	x				Tailoring Adding elements Cultural modification	Setting Population	–	x		x		x	x	x	x	x		Acceptability Adoption Fidelity	Created four new videos, one for each prevention strategy: refuse, explain, avoid and leave
Burgio, 2009 [68]	x	x	x	x	x	Tailoring Removing elements Shortening	Setting Population	Mode/medium Deliverer	x		x	x	x	x		x	x		Acceptability Adoption Fidelity Feasibility Individual satisfaction	Reduced number of home visits and shortened time span of the intervention
Fiscian, 2009 [69]	x	x	x		x	Tailoring Adding elements Removing elements Lengthening Reorder elements Integrating other approach Cultural modification	Setting Population	Mode/medium	x		x	x	x	x		x	x		Acceptability Fidelity Individual satisfaction	Modified role-play stories to use African names and settings and simplified scripts to a sixth-grade reading level
Mueller, 2009 [31]	x	x				Adding elements Lengthening Substitution Loosening structure	Setting	–	x		x	x	x	x		x			Acceptability Feasibility Sustainability Individual satisfaction	Adapted from community agency or after-school programs to be integrated into existing school curriculum
Pekmezi, 2009 [70]	x	x			x	Tailoring Cultural modification	Population	–	x		x			x	x	x	x		Acceptability Feasibility Individual satisfaction	Intervention materials and research measures were translated into Spanish through an iterative process involving both translation and back-translation
Stevens, 2009 [71]	x	x	x		x	Tailoring Adding elements	–	Deliverer		x	x	x	x	x		x	x		Fidelity	Support teams for caregivers were created
DePue, 2010 [72]	x	x	x	x	x	Tailoring Adding elements Loosening structure Cultural modification	Setting Population	Mode/medium Deliverer	x	x	x	x	x	x	x	x	x		Adoption Feasibility	Incorporated local cultural features in flipcharts, including quotes from focus groups, culturally relevant examples of healthy behaviors, local sources of stress, and effective local coping strategies
Domenech Rodriguez, 2011 [73]	x	x	x			Tailoring Adding elements Loosening structure Cultural modification	Setting Population	Deliverer	x	x	x	x	x	x	x	x	x		Acceptability Fidelity Feasibility Individual satisfaction	Sayings, or *dichos*, were incorporated generously into treatment manual as parents used them during the parent training sessions
Poulsen, 2010 [39] Vandenhoubt, 2010 [75]	x	x	x			Tailoring Adding elements Substitution Cultural modification	Setting Population Other	Mode/medium Deliverer	x	x	x	x	x	x	x	x	x	x	Acceptability Adoption Fidelity Sustainability Individual satisfaction	Owing to low literacy rates among local adults, drawings were used to illustrate messages that were originally conveyed through text on posters and handouts
Sadler, 2010 [76]	x					Tailoring Adding elements Shortening Reorder elements Cultural modification	Population	Deliverer	x					x	x				–	PowerPoint voice over changed to be in the first person instead of third to inspire comradery and motivation for women battling cancer together through clinical trials
Rotheram-Borus, 2011 [77]	x	x	x			Tailoring Adding elements Removing elements Shortening Substitution Integrating intervention Repeating elements Cultural modification	Setting Population	Mode/medium Deliverer	x					x	x	x	x		Other	The intervention content and framing was adapted to resonate with Buddhist values and idioms around “sound body and sound mind”, as well as Thai values around the importance of family and community in health and well-being
Cardona, 2009 [78]	x	x	x			Tailoring Adding elements Loosening structure Cultural modification	Setting Population	Other	x	x	x	x	x	x		x	x		Acceptability Adoption Fidelity Feasibility Individual satisfaction	Substituted a booster session with a session on “Parenting between two cultures” to add relevance to Latino immigrant families
Feinberg, 2012 [79]	x	x	x	x		Tailoring Removing elements Integrating intervention Cultural modification	Setting Population	Mode/medium Deliverer Other	x	x	x	x	x	x	x	x			Acceptability Fidelity Feasibility Individual satisfaction	Reframed the focus of the intervention from prevention of depression to learning new skills to deal with everyday stress, with an emphasis on parenting
Parker, 2012 [81] Chen, 2013 [82]	x	x	x			Tailoring Adding elements Removing elements Lengthening Reorder elements Cultural modification	Population	Mode/medium	x	x	x		x	x					Acceptability Adoption Fidelity Feasibility	Created “action plan for sustainability” to link participants with exercise/disease self-management programs in neighborhood
Reid, 2012 [83]	x	x				Tailoring Adding elements Departing Cultural modification	Setting Population	Other	x		x	x	x	x	x	x			Acceptability Adoption Fidelity Feasibility Individual satisfaction	Sociocultural norms, values, beliefs, and myths were applied to role-play scenarios and exercises
Rosati, 2012 [84]	x	x				Tailoring Adding elements Loosening structure Cultural modification	Setting	Mode/medium	x	x		x		x		x	x		Acceptability Adoption Fidelity Feasibility Individual satisfaction	Added a unit targeting adolescent dating and sexual behavior after conducting focus groups with Thai parents
Tomioka, 2012 [85]	x	x		x	x	Tailoring Adding elements Lengthening Cultural modification	Population	–	x	x	x	x		x		x	x		Acceptability Adoption Fidelity Feasibility Sustainability Individual satisfaction	Added opening session with a prayer, a 6-month reunion, and provided certificate of completion
Danielson, 2013 [33]	x	x	x		x	Tailoring Shortening Loosening structure Departing	Setting Population	Mode/medium					x	x		x	x		Adoption Fidelity Feasibility Other Individual satisfaction	Used a web-based delivery platform instead of small group sessions with 10–12 girls
Fasula, 2013 [86]	x	x	x	x		Tailoring Adding elements Shortening Lengthening Loosening structure Cultural modification	Setting Population	Deliverer	x	x	x	x	x	x	x				–	Several intervention elements were added to increase participants’ risk awareness, knowledge, and skills related to substance use, including a group discussion about the pros and cons of substance use, how drugs/alcohol contribute to sexual risk, and strategies for avoiding risk
Parker, 2013a [88] Parker, 2013b [89]	x	x	x			Tailoring Adding elements Removing elements Shortening Integrating other approach Cultural modification	Setting Population	Mode/medium	x	x	x	x		x	x	x	x		Acceptability Feasibility	Changed delivery from individual to group so there was peer reinforcement content
Wainer, 2013 [90]	x	x	x	x	x	Shortening Loosening structure	Setting	Mode/medium				x		x	x		x		Acceptability Fidelity Feasibility	Therapists completed the online training program on computers in their homes or in the research lab
Williams, 2013 [91]	x	x	x	x		Tailoring Adding elements Cultural modification	Setting Population	Deliverer	x	x	x	x	x		x	x			Adoption	The culturally adapted intervention took a more deliberate and structured approach to including the family in discussion and planning
Baydala,2014 [92]	x	x			x	Tailoring Adding elements Lengthening Cultural modification	Population	–	x	x	x	x	x	x	x	x	x		Acceptability Adoption Fidelity Feasibility Sustainability	Elders suggested inclusion of lessons that embraced Aboriginal spirituality, such as an activity on healing the worried mind where students were encouraged to take their worried mind to *Waka* (God/Creator) and engage in *wacigebi* (prayer)
Broning, 2014 [93]	x	x				Tailoring Cultural modification	Population	–				x			x	x	x		–	Intervention was translated and adapted to German culture, taking into account family-based interventions are especially culture-sensitive regarding role-model behavior, values and norms
Cariou, 2014 [94]	x	x		x	x	Tailoring Adding elements Removing elements	Setting Population	–	x	x	x	x	x	x		x	x	x	Adoption Sustainability Other	Eliminated optional poolside activities and retained the few that were feasible based on available resources
Reback, 2014 [96]	x	x	x	x	x	Tailoring Adding elements Removing elements Shortening Reorder elements Cultural modification	Setting	Deliverer	x		x	x	x	x		x	x		Adoption Feasibility Sustainability	Gay-specific cultural references were updated to maintain cultural relevancy (i.e., exchanging references to telephone dating lines with references to social networking web sites)
Riggs, 2014 [32]	x		x		x	Adding elements Shortening	–	Mode/medium Deliverer	x	x			x	x	x	x	x		Acceptability Fidelity Feasibility	Masters-level research interventionists delivered treatment rather than medical staff
Tu, 2014 [98]	x	x	x	x	x	Tailoring Removing elements	Population	Deliverer				x		x		x	x		–	In-person education from health educator was deleted

^1^Adaptation type: (1) content, (2) context, (3) delivery, (4) training, and (5) evaluation

^2^Adaptation steps: (1) community assessment, (2) selection, (3) determine level of change, (4) train staff, (5) consult stakeholders**/**experts, (6) prepare materials, (7) pilot, (8) implement, (9) evaluate, and (10) other

### Presentation of adapted elements

The authors used a variety of formats to present their adaptation processes. All 42 adaptations were described in the article’s narrative, while others also used tables and figures to present certain elements. Seventeen (40.5%) included a table of the adaptations or modifications made. Three adaptations (7.1%) illustrated the adaptation process with a figure, and two (4.8%) included a side-by-side comparison of the adapted and original EBIs.

## Discussion

This study presents findings based on a systematic review of published reports of adaptations of 42 EBIs. We present a systematic characterization of reasons for adapting EBIs, types of modifications made, steps taken during adaptation, reference to existing adaptation frameworks, and the constructs measured in evaluations of the adapted EBIs. In our review, the most common reasons for adaptation were to be relevant to a particular culture or new population, and to implement a program in a new setting. A previous study by Moore also found cultural adaptation to be a common reason for adaptations among evidence-based grantees, although less frequently (43% compared to our 64%) [15]. Higher frequency reasons in Moore’s study were related to resource constraints or logistics: lack of time (80%), limited resources (72%), difficulty retaining participants (71%), and resistance from implementers (64%) [15].

Among our included reports, all adaptation teams, or individuals involved in the research or adaptation, conducted content modifications, most commonly tailoring, adding or removing elements, and shortening. In their review of 32 published descriptions of interventions implemented in routine care or community settings, Stirman and colleagues also found the same four content modifications most frequently reported [1]. Consistent with the Stirman review, we found that context modifications were the next most frequently mentioned type of adaptation for either the program population or setting. Stirman, however, also found that format changes were frequently described [1]. In our review, delivery modifications were described in the majority of the studies, with training and evaluation modifications much less common. It is unclear whether these did not occur or were less often reported. Moore’s review found slightly different frequencies of modifications, with more reports of changes related to logistics such as changes in delivery and dose, and much less frequent content changes [15].

Like Krivitsky, we also found that 29% reported removing elements [34]. This type of adaptation should be explored more because of its implications for reducing the fidelity to program core elements and potentially reducing the EBI’s effects [35]. Additionally, four studies explicitly described changing the core elements of the original EBI. This is an area of concern because the integrity of the original program could have been jeopardized. More research is needed to understand why the elements were deleted and if the program implementers (i.e., researchers, community planners) consulted others before undertaking this change. The low reporting of changes to core elements may be because it is difficult to identify what the core elements are in an EBI. They may include elements that are readily adapted such as delivery or content. However, unless the original developers of the program or health-related online clearinghouses or resources where they are housed clearly describe them, it is often difficult for planners to identify them. Therefore, considerations of fidelity are critical when making decisions about what to adapt [36]. In a systematic review by Gearing and colleagues of 24 meta-analyses and review articles focusing on fidelity over the past 30 years, the authors identified core components of fidelity including design, training, and monitoring of intervention receipt and suggested that greater attention is needed to document threats to fidelity that remain underreported [37]. While this is true for any implementation effort, it is even more important to consider when making and reporting adaptations. Of particular note in our findings, while many authors reported changes to the delivery of the EBI, including who delivered it, there were fewer who reported adapting training or monitoring of that delivery.

Although cultural modification is not part of Stirman’s taxonomy of adaptation modifications, we found that almost 75% of the authors described their adaptation in this way. Because cultural adaptations would almost always require some adaptation related to population and context, it is likely that authors in the Stirman review reporting adaptations to content, context, and new populations were, at least in some cases, making cultural adaptations. More clarity in definitions of what is meant by each type of adaptations is needed.

Our review uses a new taxonomy of steps or tasks for adaptation derived from a scoping study of existing frameworks [23]. We looked for nine steps or tasks and found that two adaptations reported all of the steps [38, 39], with the mean number being seven. Thus, most adaptation teams completed the majority of the steps. Overall, we found that most reported community assessment, preparation of materials, implementation, evaluation, and engaging stakeholders/experts as part of a program planning process. The least common step was selection of the EBI. This may be because some program staff may have already decided on the EBI a priori and did not undertake a process to review candidate EBIs and select one.

The Escoffery classification from a review of adaptation frameworks seems applicable to real-world adaptation and could be used by others as a taxonomy for describing adaptation steps [23]. However, there are details that may be nuanced that are important to understand for the field both in describing adaptation steps and for informing future adaptations of the same EBI. For example, some reports include information about which components of the intervention were pilot tested and what decisions were made based on assessment finding, who the stakeholders were, and how they were engaged. Additionally, specific details about who is involved in the adaptation process (stakeholders, target population, program deliverers, health promotion, and behavioral scientists) and who makes the final decisions on what changes to make are critical to document. This information could be very important in interpreting reasons for specific adaptations and informing subsequent ones for future EBI implementation.

Capacity building efforts can assist practitioners to document the process in more detail and be deliberative or proactive with adaptations. The Cancer Prevention and Control Research Network (CPCRN) has modules on program selection of EBIs and adaptation with tools that help practitioners to document the discussion and decisions related to those processes in their Putting Public Health Prevention into Practice training [40]. In addition, the new online decision support tool, IM ADAPT, walks public health professionals through a systematic process to create a logic model for the adapted EBI and a selection adaptation, implementation, and evaluation plan based on intervention mapping [41, 42].

Among those reports that included an evaluation, the most common outcomes were acceptability, fidelity, and feasibility. This is not surprising since acceptability and feasibility of an intervention is often associated with program adoption [27]. Only one third reported the use of an adaptation framework to inform their process. This number is surprisingly low. Adaptation frameworks would provide guidance and rationale for this process and should be used. Many frameworks exist, but perhaps program planners may not be aware of them [34] or may not know how to follow them without training or technical assistance. Due to the limited research on program adaptation, there also may not be the emphasis on adaptation models and frameworks. There needs to be increased dissemination and education on these frameworks to offer assistance with recommended steps in program adaptation.

### Implications

Through a search of the published literature, this is the first systematic review of adapted evidence-based public health interventions internationally. Findings from the present study lead to important implications for the field of implementation science. First, many of the reasons for adaptation focus on either a change of population or setting, while the most common modifications were related to content, context, and delivery. Program developers of EBIs could anticipate program adaptation, instead of only adoption, and provide technical guidance in making modifications in their implementation protocols (or facilitator’s manual) or program website. Recognizing that it is likely that successful programs will be adapted, program developers should also provide guidance about the theory and mechanisms of change that were used in the intervention and where possible design flexibility to match various contexts and populations [43]. They also can serve as expert consultants to help in the adaptation process as recommended as part of adaptation steps in adaptation frameworks [11, 16, 44] or our scooping study [23]. In addition, they could support a community of their EBI adopters by making adapted versions available or offering contacts for practitioners implementing the same program. Due to the low reporting of use of adaptation frameworks, the frameworks could be more widely disseminated to inform future adaptation efforts.

Research on best methods to document program adaptation is warranted to better understand whether it is best to describe and code adaptations based on document reviews of adaptation plans, published articles or reports, interviews of the adaptation team, or all of the above. Each of these methods has limitations, but implementing them all may not be practical for research studies. Finally, we found a variety of styles in reporting the reasons, modifications made, and process of adaptation. Standardization of reporting elements on program adaptation would guide professionals in describing their changes to EBIs and advance the field. Through this process of better reporting on adaptations, practitioners and program planners can better understand the reasons for adaptation, the adaptation process, and results to inform their own practice. Currently, TREND and Standards for Reporting Implementation Studies (StaRI) statement only ask researchers to report on adaptation in general or adaptation results [45, 46]. Other critical elements of adaptations that we have identified (i.e., reasons, types of adaptations, steps taken) are not mentioned or delineated. There is a growing body of literature of adaptation taxonomies that could be recommended for some of these elements, including types of modifications [1, 47], reasons, timing and valence [15, 47], frameworks employed, and steps taken [23]. Creation of detailed reporting standards for program adaptation will result in commonalties for describing adaptations in the published and gray literature and will advance the field of implementation science in terms of producing adaptation data for further analysis.

Future research could explore planned versus unplanned adaptations and patterns of program modifications and the reasons for that happening. We present some early findings of patterns of modifications made to public health EBIs, but there is scarce understanding of them. In addition, further evaluations of adapted interventions are required to determine if adapted versions are as effective as the original program or other adapted versions. In this study, over 60% of the adaptation reports were non-experimental (i.e., observational, pilot program, post-test only) and less than one third were experimental. It is important for the field to have more rigorous evaluations of adapted programs to understand their outcomes and if their effects are comparable, better, or worse than the original EBI. Some preliminary research suggests that adapted versions of interventions are not associated with worst outcomes [48]. The evaluations also could inform if different types or combinations of modifications (e.g., content and context) impact effectiveness as well. Researchers also should determine critical adaptation elements to record and standardize across studies. Finally, while there have been repositories of evidence-based programs for public health practice such as the National Registry for Effective Programs and Practices [49] and Research Tested Intervention Programs (RTIPs) [50], there is no clearinghouse for adapted programs for the field to understand the issues around external validity of EBIs. Chambers recommends the creation of an adaptome to catalog adapted programs and their results to share with the field to potentially address this gap [51].

Several limitations exist for this study. Although we searched for relevant articles of adapted EBIs, it is likely that some articles were overlooked based on our search strategy. For example, we did not review gray literature for adapted EBIs. In addition, we limited our studies to those that focus on public health interventions and excluded clinical interventions. Additionally, our data on adaptations made and outcomes were limited to the authors’ description in the article and were not augmented with other data (e.g., surveys of authors). Although we had two raters to increase the reliability of the data abstracted, some of the modifications may have been underreported if the authors fail to report on that aspect (i.e., training) or may not have fully implemented the program yet (i.e., evaluation). Finally, while our review included adapted interventions globally, we did not review articles in languages other than English. However, we were able to find 16 studies in international settings. A limitation of this study is that we did not confirm with the authors that all of the adaptations made were reported; for example, some could have not reported on unplanned adaptation since some were not yet implemented. It is helpful for planners to document all adaptation, both planned and unplanned [52], for other practitioners to learn from this process. Finally, this review is becoming dated, especially in an area with a much active research and reporting.

## Conclusion

This review offers a practical examination of adaptation across multiple programs and program types that were  implemented in community settings. It reports systematically on reasons for adaptation, types of modifications, and steps of adaptations for public health EBIs in public health practice. Adaptations are occurring in natural settings for a variety of reasons, and commonly, adaptations are made to intervention content or context. A few steps were used across adaptation teams in the process of adaptations, but the science of adaptation is still an emerging area of study in implementation science. More critical appraisal of intervention adaptations and their outcomes could assist with EBI transferability to increase the scale up and spread of EBI to increase population health impact.

## Additional files

Additional file 1: Systematic review search terms. (DOCX 13 kb)

Additional file 2: Abstraction categories and definitions. (DOCX 19 kb)

## Abbreviations

CPCRN: Cancer Prevention and Control Research Network
EBIs: Evidence-based interventions
MeSH: Medical Subject Headings
