# Supplementary material for: A systematic review of adaptations of evidence-based public health interventions globally
Source: Implement Sci. 2018 Sep 26;13:125. doi: 10.1186/s13012-018-0815-9 (PMC6158804; doi:10.1186/s13012-018-0815-9)
Supplement: Supplementary file 1 — Systematic review search terms. (DOCX 13 kb) [file 13012_2018_815_MOESM1_ESM.docx]

Additional file 1. Systematic Review Search Terms

| 1. Adaptation AND Evidence Based Practice AND Public Health Practice AND Health Behavior 2. Adaptation* AND Evidence Based Practice AND Public Health Practice AND Health Behavior 3. Adapt* AND Evidence Based Practice AND Public Health Practice AND Health Behavior 4. Adapt* AND Evidence Based Practice AND Public Health Practice 5. Adaptation AND Evidence Based Practice AND Public Health Practice 6. Quality of Health Care AND Adapt* AND Health Behavior AND Public Health Practice 7. “Adaptation” AND Evidence Based Practice AND Public Health Practice AND Health Behavior 8. “Adaptation” AND Evidence Based Practice AND Public Health Practice AND Health Behavior AND Quality of Healthcare |
| --- |
