# Supplementary material for: A systematic review of adaptations of evidence-based public health interventions globally
Source: Implement Sci. 2018 Sep 26;13:125. doi: 10.1186/s13012-018-0815-9 (PMC6158804; doi:10.1186/s13012-018-0815-9)
Supplement: Supplementary file 2 — Abstraction categories and definitions. (DOCX 19 kb) [file 13012_2018_815_MOESM2_ESM.docx]

Additional file 2. Abstraction Categories and Definitions

| **Abstraction Category** | **Definition** |
| --- | --- |
| **Type of Modification (Stirman et al., 2013)** | |
| **Content** | Modifications made to the materials/content itself, or that impact how aspects of the treatment are delivered. |
| 1) Tailoring | “Any minor change … that leaves all the major intervention principles and techniques intact while making the intervention more appropriate, applicable, or acceptable (e.g., modifying language, creating slightly different version of handouts … cultural adaptations).” |
| 2) Adding elements | “Additional materials or activities … that are consistent with the fundamentals of the intervention (e.g., adding role play exercises to a unit on assertiveness in a substance abuse prevention intervention, adding new disease topics).” |
| 3) Removing elements | “Particular elements of the intervention are not included (e.g., leaving out a demonstration on condom use in an HIV prevention intervention for adolescents).” |
| 4) Shortening | “[Less] … time than prescribed is used to complete the intervention/ session(s) (e.g., shorter spacing between sessions, or shortening sessions, … fewer sessions, or going through particular modules or concepts more quickly without skipping materials).” |
| 5) Lengthening | “[More] … time than prescribed … is spent to complete the intervention or … sessions (e.g., greater spacing between sessions, longer sessions, more sessions, or spending more time on one or more modules/activities or concepts).” |
| 6) Substitution | “A module or activity (e.g., game) is replaced with something that is different in substance (e.g., replacing a module on condoms with one on abstinence …).” |
| 7) Reorder elements | “Modules/activities or concepts are completed in a different order from what is recommended … This code would not be applied if the protocol allows flexibility in the order … ” |
| 8) Integrating other approach | “The intervention of interest is used as the starting point, but aspects of different therapeutic approaches or interventions are also used (e.g., integrating an ‘empty chair’ exercise into a ‘CBT for Depression’ treatment protocol).” |
| 9) Integrating intervention | “Another intervention is used as the starting point, but elements of the intervention of interest are introduced (e.g., integrating motivational enhancement strategies into a weight loss intervention protocol).” |
| 10) Repeating elements | “One or more modules, sessions, or activities that are normally prescribed or conducted once during a protocol are used more than once.” |
| 11) Loosening structure | “Elements intended to structure intervention sessions do not occur as prescribed in the manual/ protocol (e.g., the ‘check-in’ at the beginning of a group intervention is less formally structured; clinician does not follow an agenda that was established at the beginning of the session).” |
| 12) Departing | “The intervention is not used in a particular situation or the intervention is stopped, whether this stoppage was for part of a session or a decision to discontinue the intervention altogether (e.g., ‘this client was so upset that I just spent the rest of today’s session letting him talk about it instead of addressing his health behaviors’).” |
| **Cultural** | Authors mention adapting the intervention to make it more culturally appropriate, with or without referencing a specific framework.[2] |
| **Context** | Modifications made to the setting or population. |
| 1) Setting | “Intervention is being delivered in a different location or setting (e.g., a treatment originally designed to be used in a mental health clinic setting that is now delivered in primary care).” |
| 2) Population | “An intervention that was specifically developed to target a particular population is being delivered to a different population than originally intended (e.g., an intervention developed for patients with Borderline Personality Disorder is now being delivered to individuals with Substance Dependence).” |
| **Delivery** | Modifications made to the mode/medium of program delivery or to the implementers/deliverers. |
| 1) Mode/Medium | “Changes are made to the format or channel of treatment delivery (e.g., a treatment originally designed to be used one-on-one that is now delivered in a group format, a change from print to tech-based medium).” |
| 2) Deliverer | “The intervention is being delivered by personnel with different characteristics (e.g., a treatment originally designed to be administered by a mental health professional is now delivered by clergy).” |
| **Training** | **Modifications made to the procedures for training personnel.** |
| **Evaluation** | **Modifications made to the procedures for evaluating the program.** |
| **Core Elements** | **Describes any changes to the core elements,** **i.e.,** **“critical features of an intervention’s intent and design [that] are thought to be responsible for its effectiveness.” [Eke] This includes changes to any identified intervention components whether or not they were specifically called “core elements”.** |
| **Reason for Adaptation** | **Stated reason(s) for adaptation, e.g., generate or maintain engagement; strengthen/reinforce message; material addresses local context, reach special/specific audience; increase fit.** |
| **Implementers** | **The individual or groups who decided how to modify the intervention and/or the process of adaptation (e.g., meetings, focus groups with participants, stakeholders).** (Stirman et al, 2013) |
| **Steps in Adaptation** |  |
| 1) Community Assessment | Conduct focus groups/needs assessment with the new target population or elicitation interviews with the key stakeholders |
| 2) Selection | Agency makes a final selection on the EBI using assessment data. Selection also involves building capacity with subject matter and implementation skills and consulting with the community and staff regarding the decision. |
| 3) Determine Level of Change | “Determine what changes are needed based on the assess action step assessments…which uncover gaps where the intervention should be adapted, where the agency needs to build capacity, and areas where the agency could benefit from collaborating with technical assistance (TA) providers and partners.” [Bartholomew Eldredge et al.] |
| 4) Train Staff | “Training personnel, including: 1) facilitators in group management and facilitation skills, 2) recruiters and retention staff in effective recruitment and retention techniques, 3) assessment staff in administering study assessments, and 4) data management staff in managing study data.” [Wingood & DiClemente] |
| 5) Consult Experts | E.g., “identify experts who could serve as consultants to provide specific content expertise.” (e.g., identify topical experts knowledgeable about the disease or the target audience for the intervention). [Wingood & DiClemente]  “Work with an expert on the core/internal logic of the EBI.” [Bartholomew Eldredge et al.] |
| 6) Prepare Materials | E.g., “integrate content from needs assessment data (i.e., from topical experts) based on capacity of agency and create draft 2 of adapted EBI, and integrate scales that assess new intervention content in study survey.” [Wingood & DiClemente] |
| 7) Pilot | “Pre-test materials with community advisory board, get feedback, and revise if necessary and pre-test materials with members of the target audience, get feedback, and revise as necessary until adapted materials work.” [Bartholomew Eldredge et al.] |
| 8) Implement | “Implement adapted intervention (most of the adaptation has been systematically completed).” [Bartholomew Eldredge et al.] |
| 9) Evaluate | “Collect process measures on adapted intervention implementation, conduct process monitoring and evaluation on adapted intervention implementation, collect intervention outcome measures, conduct outcome monitoring and evaluation, and make small changes as needed to staff and intervention based on process evaluation findings.” [Bartholomew Eldredge et al.] |
| **Consulted with Developer** | Authors or adaptation team communicated with the original program developer(s). |
| **Implementation Outcomes** | Definitions from Proctor et al., 2011 |
| 1) Acceptability | “The perception among implementation stakeholders that a given treatment, service, practice, or innovation is agreeable, palatable, or satisfactory (e.g., in terms of content, complexity, comfort, delivery, and credibility).” |
| 2) Adoption | “The intention, initial decision, or action to try or employ an innovation or evidence-based practice.” |
| 3) Fidelity | “The degree to which an intervention was implemented as it was prescribed in the original protocol or as it was intended by the program developers.” |
| 4) Feasibility | “The extent to which a new treatment, or an innovation, can be successfully used or carried out within a given agency or setting.” |
| 5) Sustainability | “The extent to which a newly implemented treatment is maintained or institutionalized within a service setting’s ongoing, stable operations.” |
